# Meiotic Drive Impacts Expression and Evolution of X-Linked Genes in Stalk-Eyed Flies

**DOI:** 10.1371/journal.pgen.1004362

**Published:** 2014-05-15

**Authors:** Josephine A. Reinhardt, Cara L. Brand, Kimberly A. Paczolt, Philip M. Johns, Richard H. Baker, Gerald S. Wilkinson

**Affiliations:** 1 Department of Biology, University of Maryland, College Park, Maryland, United States of America; 2 Department of Biology, University of Rochester, Rochester, New York, United States of America; 3 Bard College, Annadale-on-Hudson, New York, United States of America; 4 Sackler Institute for Comparative Genomics, American Museum of Natural History, New York, New York, United States of America; University of California, Berkeley, United States of America

## Abstract

Although sex chromosome meiotic drive has been observed in a variety of species for over 50 years, the genes causing drive are only known in a few cases, and none of these cases cause distorted sex-ratios in nature. In stalk-eyed flies (*Teleopsis dalmanni*), driving X chromosomes are commonly found at frequencies approaching 30% in the wild, but the genetic basis of drive has remained elusive due to reduced recombination between driving and non-driving X chromosomes. Here, we used RNAseq to identify transcripts that are differentially expressed between males carrying either a driving X (X^SR^) or a standard X chromosome (X^ST^), and found hundreds of these, the majority of which are X-linked. Drive-associated transcripts show increased levels of sequence divergence (dN/dS) compared to a control set, and are predominantly expressed either in testes or in the gonads of both sexes. Finally, we confirmed that X^SR^ and X^ST^ are highly divergent by estimating sequence differentiation between the RNAseq pools. We found that X-linked transcripts were often strongly differentiated (whereas most autosomal transcripts were not), supporting the presence of a relatively large region of recombination suppression on X^SR^ presumably caused by one or more inversions. We have identified a group of genes that are good candidates for further study into the causes and consequences of sex-chromosome drive, and demonstrated that meiotic drive has had a profound effect on sequence evolution and gene expression of X-linked genes in this species.

## Author Summary

Sex chromosome meiotic drive causes changes in the sex-ratios of natural populations, and may even lead to extinction if the driving element reaches high frequency. However, very little is known about the genes that cause sex-ratio drive, and no causal gene has been identified in a species that consistently exhibits distorted sex ratios in natural populations. Several species of stalk-eyed flies in southeast Asia – genus *Teleopsis* – express X chromosome drive, but the genes underlying drive have been difficult to locate due to reduced recombination between drive and standard X chromosomes presumably caused by the presence of a large inversion. Here, we use high throughput RNA sequencing to identify over 500 transcripts that are differentially expressed in the testes due to the effects of a driving X chromosome (X^SR^) in *T. dalmanni*. Most of these are X-linked, evolve more rapidly than control genes, and exhibit elevated expression in the gonads. Finally, X^SR^ has become genetically differentiated from standard X chromosomes – using the RNA sequence data, we found nearly 1000 sites in X-linked transcripts and only a handful in autosomal transcripts where there was a fixed nucleotide difference. We conclude that X^SR^ has led to widespread sequence and expression divergence on the X chromosome in *T. dalmanni*.

## Introduction

Meiosis typically results in an equal transmission probability of each allele from parent to gamete. This seemingly cooperative outcome masks an inherent genetic conflict. Alleles on any one chromosome would increase in frequency more rapidly if that chromosome passed to all, instead of half, of the gametes produced. Such selfish alleles cause meiotic drive and would be expected to sweep quickly to high frequency or even fix. Detecting autosomal drive is difficult because distorted segregation patterns of chromosomal markers must be observed. However, when a drive allele is on a sex chromosome, the sex ratio of offspring is distorted. As a driving sex chromosome increases in frequency, the sex ratio in the population will become increasingly biased. If a drive allele nears fixation, population extinction due to absence of the rare sex is expected [1]. Alternatively, because the rare sex will have a fitness advantage [2], alleles which act to restore the sex ratio to equality will be favored. Potential mechanisms to counter fixation of drive alleles include sexual selection in which standard males outcompete drive males in mating or sperm competition [3]–[5], selection acting on female X^SR^ carriers [6], and the evolution of loci on the other sex chromosome or autosomes that suppress drive [1].

Genomic conflicts in general, and meiotic drive in particular, can create dynamic evolutionary systems that influence patterns of molecular evolution and the evolution of gene expression. Drive loci have a strong local fitness advantage, but decrease fitness of the population because selection cannot act efficiently to remove low-fitness drive carriers [7]. In addition, many examples of suppressed or “cryptic” drive systems have been uncovered in Drosophila in which either autosomal or Y-linked suppressors mask the phenotypic expression of the drive allele in extant populations [8]–[13]. Like the drive locus, at the time they arose, these loci would have been strongly selected, whether or not they provided any benefit to the organism [14]. Furthermore, the inherent fitness advantage of drive and suppressor alleles is expected to lead to strong effects on linked neutral polymorphism as these alleles increase in frequency - as has been documented for both autosomal [15], [16] and sex-ratio drive [17] in Drosophila species. In fact, the theoretical effects of meiotic drive on the genome are so extreme that it has been invoked as a possible cause for fundamental phenomena [18], such as homologous recombination [19] and Haldane's Rule [20]–[22], with some experimental evidence of the latter [23], [24].

Although meiotic drive has been observed in many different species, particularly dipterans (reviewed in [14]), the genetic basis of drive is known in only a few cases, and of these, none distort sex ratios appreciably in natural populations. Partly, this is due to the tendency of actively driving loci to be found on sex-ratio X chromosomes that do not recombine with standard X chromosomes due to the presence of one or more inversions. This has prevented fine mapping of the drive loci in most cases [25], [26]. Intriguingly, both cases of X-chromosome drive that have been mapped to the gene level are associated with copy number variants and occur in *Drosophila simulans*. The “Paris” sex-ratio drive system (also known as X^SR6^) recombines freely and populations polymorphic for both the suppressor and the driver exist [8], allowing genetic dissection using interpopulation crosses. The element that causes drive has been mapped to a segmental duplication of six genes on the X chromosome, with associated changes in gene expression for some of the duplicated genes [27]. The “Winters” drive system is caused by an X chromosome drive gene, *Dox*, which is an imperfect duplication of a previously existing gene, *MDox*, and is suppressed by an autosomal retroduplicated gene, *Nmy*, which functions to silence *Dox* through an RNAi-like mechanism [10], [11].

In the stalk-eyed fly, *Teleopsis dalmanni*, males carrying a meiotic drive X chromosome (X^SR-1^ or X^SR-2^) [28] parent mostly daughters [29]. Drive chromosomes are present in natural populations but appear not to recombine with standard X chromosomes in laboratory crosses [26]. The X chromosome gene content in *T. dalmanni* is mostly orthologous to Muller element B, i.e. chromosome arm 2L in *Drosophila melanogaster* [30]. Thus, the genetic context for X chromosome drive in Teleopsis is distinct from that found in the Drosophila systems described above. Furthermore, meiotic drive associates with a number of characters that influence male reproductive success, including eye-stalk length [26], [31], sperm precedence [32] and sperm morphology [33]. The fate of the drive allele may be influenced by sexual selection acting against the drive X chromosome [34], which also causes males to have shorter than average relative eye-stalk length [26], [35]. Conversely, females carrying a drive X chromosome may have elevated fecundity, providing a possible explanation for why drive X chromosomes are not lost or suppressed [28]. However, the genetic basis for most of these traits - and meiotic drive itself - is unknown apart from the association with X^SR^. Like many chromosomes carrying meiotic drive loci, X^SR^ – or at least the portion of X^SR^ that causes both meiotic drive and associated phenotypic differences – does not recombine with standard X chromosomes [26], making identification of causal loci difficult.

To identify genes that are involved in sex-ratio drive and associated phenotypes in *T. dalmanni*, we performed RNAseq on replicate pools of testes carrying meiotic drive (X^SR^) and standard (X^ST^) X chromosomes. We aligned these reads to a transcriptome assembled *de novo*, identified hundreds of transcripts differentially expressed between X^SR^ and X^ST^ testes, identified their expression patterns, determined whether they were X-linked, Y-linked or autosomal, and identified fixed differences between the two samples. We found that drive-associated transcripts were more likely to be X-linked and to have elevated expression in testes (as expected) as well as in both testes and ovaries. These transcripts were also more rapidly evolving than a control set and included a number of interesting candidate genes with Drosophila orthologs involved in potentially relevant molecular and biological processes. Finally, we found that hundreds of X-linked transcripts carry fixed differences between X^SR^ and X^ST^ samples while only a handful of such differences were found in autosomal transcripts. Our data support previous studies [36], [37] suggesting that the X^SR^ haplotype is evolving independently from X^ST^, and reveal a group of candidate genes that will be useful targets for future studies of meiotic drive in this species.

## Results

### Differential Expression between X^SR^ and X^ST^ Testes

We sequenced RNA collected from replicate pools of testes dissected from *T. dalmanni* – Gombak males that carried the sex-ratio meiotic drive X (X^SR^) or the standard X (X^ST^) chromosome (X^SR^ and X^ST^ status was determined by microsatellite haplotype following Wright 2004 [36] and Wilkinson 2006 [28]). Reads were aligned to the *T. dalmanni* transcriptome (see methods) with bwa [38] and raw read counts were corrected using RSEM [39] to account for hits to multiple isoforms (contigs) making up the same transcript. We then used DESeq [40] with the corrected read counts to find transcripts that were differentially expressed between X^ST^ and X^SR^ testes using a FDR<0.001 cutoff and after removing transcripts which had no expression in any of the four samples. We found a total of 513 transcripts to be significantly differentially expressed between transcriptomes from X^SR^ and X^ST^ testes. As a group, we refer to these as “drive-associated transcripts”. Among them, 233 were expressed at a higher level in X^SR^ males and 280 were expressed at a lower level in X^SR^ males (Table 1). A total of 113 transcripts exhibited more than 10-fold differential expression between X^SR^ and X^ST^. For technical reasons, transcripts that are significantly differentially expressed are more likely than other genes to be expressed at a high level. In order to prevent weakly expressed transcripts from biasing our results, we defined a control gene set from among the remaining transcripts by removing the most weakly expressed genes from consideration (see Methods). We next aligned predicted proteins to the Drosophila proteome to identify putative Drosophila orthologs (Table 1). Among the drive-associated transcripts, 28.2% had putative Drosophila orthologs (18.4% among control genes). Of the remaining transcripts, 239 contain a long open reading frame, and may be *Teleopsis*-specific proteins, whereas 129 had short (<50 AA) open reading frames and may be noncoding RNA genes. Compared to drive-associated transcripts, a larger proportion of the control transcripts had short (<50 AA) open reading frames (52.3% vs. 25.1%, χ^2^ = 56.88, P<4.625 e-14, Table 1). Given that noncoding RNA genes are thought to be more narrowly and weakly expressed compared to protein-coding genes [41], [42], we speculate that an excess of presumptive noncoding RNA genes in the control gene set may be caused by the observation that drive-associated transcripts tend to be expressed more strongly than the average transcripts. Alternatively, protein-coding genes may be more likely to become drive-associated than noncoding RNA genes. Finally, we used quantitative RT-PCR to confirm differential expression of drive-associated transcripts (Table S1). After excluding weakly expressed samples, 11 of 11 transcripts replicated the qualitative pattern observed in the RNA-seq data (i.e. differentially expressed in the same direction).

**Table 1:** Characteristics of drive-associated transcripts.

	Up in X^SR^ testes		Down in X^SR^ testes		Drive-associated		Control	
Total count	233	Proportion	280	Proportion	513	Proportion	50098	Proportion
Drosophila orthologs	73	0.313	72	0.257	145	0.283	9244	0.185
Longest ORF>150 AA	31	0.133	39	0.139	70	0.136	2553	0.051
Longest ORF>50 AA, <150 AA	62	0.266	107	0.382	169	0.329	12082	0.241
No ORF>50 AA	67	0.288	62	0.221	129	0.251	26219	0.523
>5-fold differentially expressed	76	0.326	98	0.350	174	0.339	NA	NA
2-fold to 5-fold differentially expressed	82	0.352	98	0.350	180	0.351	NA	NA

### Drive-Associated Transcripts Are Enriched in Gonads

**Figure 1:**
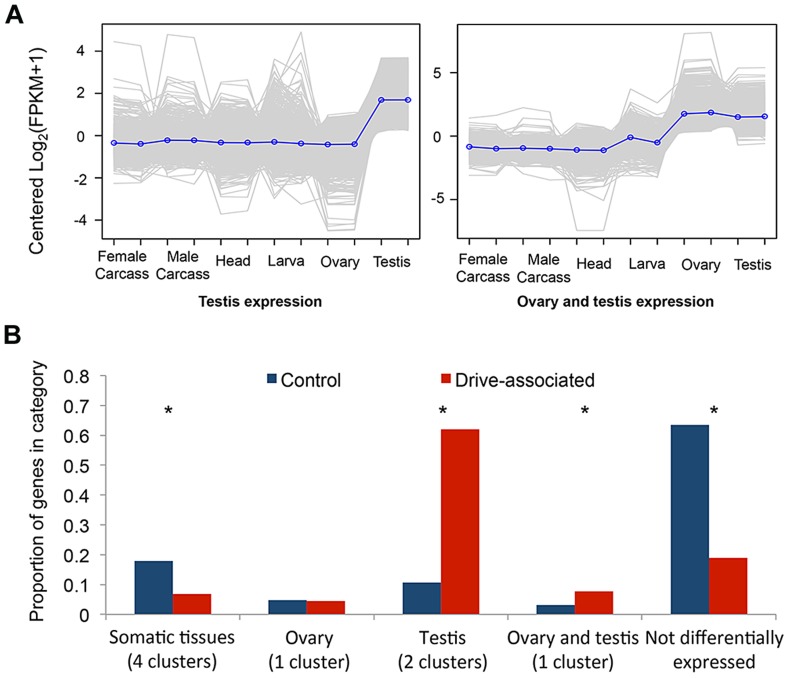
Drive-associated transcripts are enriched in the gonads. (A) K-means clustering was used to establish qualitatively distinct expression patterns of transcripts across the six sequenced tissues; clusters enriched for transcripts expressed predominantly in testes or in ovary plus testes are shown. Each grey line represents expression of a single transcript, and the blue line is the mean across all transcripts in the cluster. (B) Compared to control transcripts, drive-associated transcripts were more likely to have a significant expression pattern (categories other than “Not differentially expressed”), and in particular were enriched for two clusters associated with expression in the testis. Drive-associated transcripts were also enriched in a small cluster of transcripts expressed in both the ovary and the testes but not ovary alone. Control transcripts were more likely than drive-associated transcripts to be present in one of the somatic expression clusters (head, larvae, and adult carcass and larvae).

We performed a multi-tissue expression analysis of drive-associated and control transcripts using RNAseq from six *T. dalmanni* tissues using tools provided on the trinity website (trinityrnaseq.sourceforge.net [43]). We clustered differentially expressed transcripts to identify the eight most common patterns of gene expression and compared the number of transcripts assigned to each cluster for drive-associated and control transcripts (Figure 1A, Figure S1). Control transcripts were more likely than drive-associated transcripts to have no significant pattern of differential expression (“Not differentially expressed”), possibly because many of them could be housekeeping genes. Testes-associated clusters were enriched among drive-associated transcripts compared to controls (χ^2^ = 737.3, P<2.2 e-16). We also assessed testes-specificity in drive and control transcripts by calculating the Tau metric [44] and found more drive-associated than control transcripts were testes-specific (57.5% vs 16.9%, χ^2^ = 622.7, P<2.2 e-16). This is not surprising considering the comparison was between testes from X^SR^ and X^ST^ males. However, we also expect that a subset of drive-associated transcripts are likely to be involved directly in various aspects of spermatogenesis, given that meiotic drive affects sperm development in *T. dalmanni* [26] and a closely related species [45]. Among the other expression categories, a cluster showing elevated expression in the gonads of both sexes was also enriched (Figure 1B, χ^2^ = 30.5, P<2.2 e-16), raising the possibility that genes with pleiotropic effects on female reproduction may be differentially expressed on X^SR^. Early models of sex chromosome drive predicted that drive loci could be maintained if they also cause increased fitness in heterozygous females [6], [46]. Given that gonad expressed genes are often tissue specific, it has been thought unlikely that a single gene would do both, but given that an excess of drive associated genes show elevated expression in both ovary and testis, perhaps some of these genes are involved in both increased female fecundity (see [28]) and meiotic drive.

### Drive-Associated Transcripts Are Predominantly X-Linked

**Figure 2:**
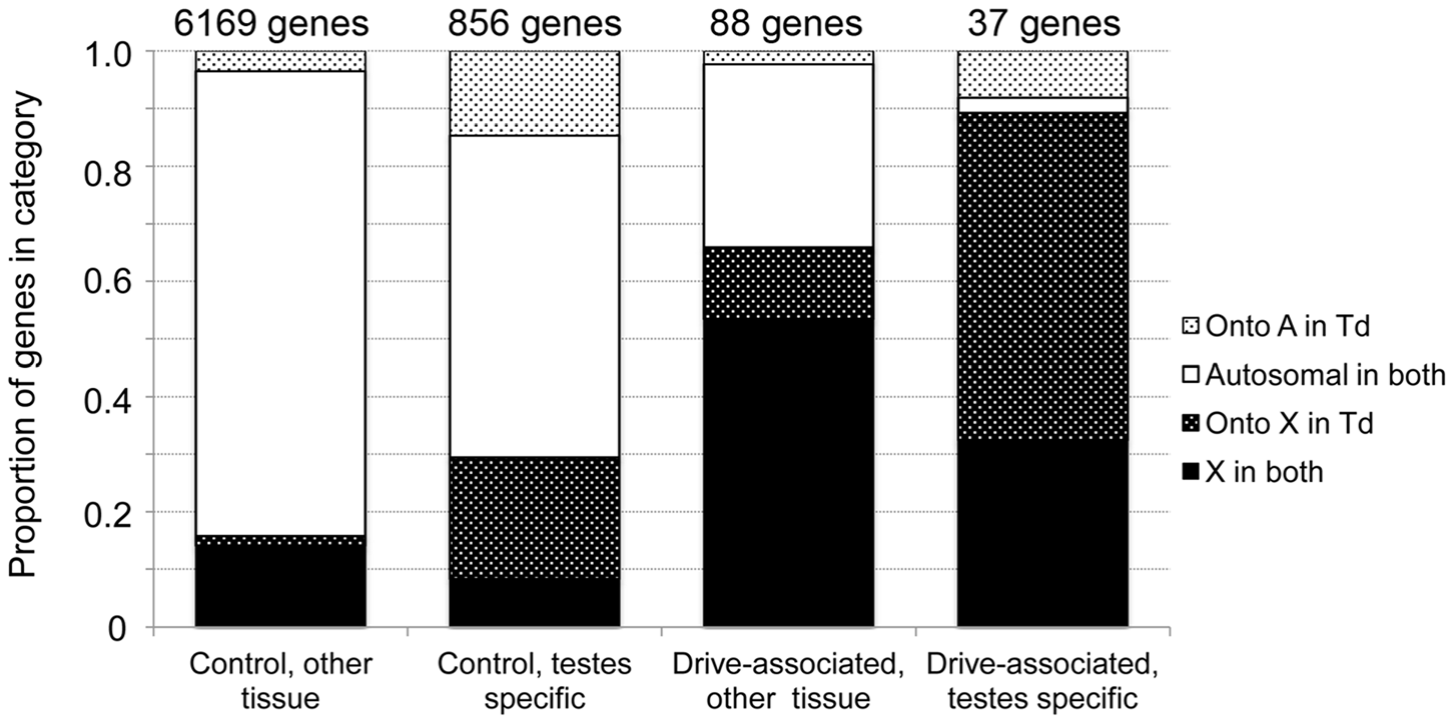
Drive-associated transcripts show an excess of X-linkage. Linkage of drive-associated and control genes determined from a comparative genomic hybridization experiment and evidence of gene movement inferred from the chromosomal locations of *D. melanogaster* orthologs. Based on previous analysis, X-linked genes in *T. dalmanni* are expected to be on 2L in *D. melanogaster*, so genes breaking this rule have moved in one lineage or the other. Solid bars indicate genes that have not moved: either they are 2L in *D. melanogaster* and X-linked in *T. dalmanni* (solid black) or not on 2L in *D. melanogaster* and autosomal in *T. dalmanni* (solid white). Stippled bars indicate genes that have moved, either onto the X in *T. dalmanni*/off of 2L in *D. melanogaster* (dark stippled) or onto an autosome in *T. dalmanni*/off of a non-2L chromosome in *D. melanogaster* (light stippled). Drive-associated transcripts are overwhelmingly X-linked especially if they are testes-specific (compare dark bars to light bars).

The presence of an X^SR^ haplotype in a male *T. dalmanni* is sufficient to cause him to parent strongly female-biased broods, regardless of his genetic background [26]. We determined whether this strong X effect extended to the level of gene expression by comparing the chromosomal linkage of drive-associated transcripts and a control set using data from a comparative genomic hybridization experiment. We found that drive-associated transcripts were strongly enriched on the X chromosome compared to the control set (78% vs 18%, χ^2^ = 256, P<2.2 e-16), suggesting that the majority of downstream effects of X^SR^ on gene expression are in *cis* rather than in *trans* (Figure 2). While the previous observation [30] that the *D. melanogaster* 2L, i.e. Muller element B, is orthologous to the *T. dalmanni* X generally holds (across all transcripts, 9.3% violate this rule), a large proportion of drive-associated transcripts (21.6%) have moved onto the X chromosome, in contrast to only 3.3% of controls (Figure 2). In *D. melanogaster*, male-specific genes have a tendency to move off of the X [47], [48], though young male-biased genes may be enriched on the X [49]. As the X chromosome in *T. dalmanni* is distinct from the *D. melanogaster* X, it is unclear whether the same pattern would be expected. While the number of moving drive-associated transcripts appears to be in large excess, drive-associated transcripts are more likely to be on the X chromosome than are controls, and much of the movement can be explained by the effect of linkage in that more genes are moving onto the X chromosome – relative to *D. melanogaster* - in *T. dalmanni* than are moving onto the autosomes (19.2% of controls and 29.7% of drive-associated transcripts have moved onto the X in *T. dalmanni*, relative to *D. melanogaster*). In addition, we recently found that in *T. dalmanni*, an excess of testes-specific transcripts have moved onto the X chromosome (unpublished data), and an excess of drive-associated transcripts are testes-specific. Indeed, among testes-specific transcripts, 21.1% of controls and 56.8% of drive-associated transcripts have moved onto the X chromosome from Muller elements other than B (Figure 2). Given these factors may be confounding, we fit nominal logistic models to predict gene movement by chromosome linkage (A or X), drive association (drive-associated/control), tissue source (testes or other) and interactions among these three factors for 7,150 transcripts. We compared three models with different interaction terms and chose the model with the lowest AICc score (Table S2, 4-parameter model). The best-fitting model explained 20.7% of the variation in gene movement (χ^2^ = 916, d.f. = 4, P<0.0001) with strong effects of X-linkage, tissue, and the interaction between X-linkage and tissue (all P<0.0001, Table S2) but no significant effect of drive-association (P = 0.1745). Therefore, while the large proportion of drive-associated transcripts moving onto the X is striking, this is most likely not due to the effect of drive *per se*. Instead, we conclude that most of the effect of X^SR^ on expression is due to genes on the X chromosome, regardless of whether they moved there recently or have persisted on Muller element B since the divergence of genus Drosophila and Teleopsis. In addition, a group of five drive-associated transcripts was found to be Y-linked (Table 2). While the number of Y-linked genes does not exceed expectation, they are of interest as potential targets of sex-chromosome drive. During spermatogenesis in drive-carrying *T. dalmanni*, the Y-bearing sperm do not complete elongation. While the genetic cause of this is unknown, in other cases of X chromosome drive the Y chromosome is the direct target of drive. For example, in the *Slx/Sly* system in mice, expression of an array of Y-linked genes is modified by the presence of a driving X chromosome [50]. Currently, we have very little information about these Y-linked transcripts. They lack *D. melanogaster* orthologs, though two of the genes appear to be protein-coding and have orthologs in the sister species *T. whitei*.

**Table 2:** Fixed differences between X^ST^ and X^SR^ are overwhelmingly X-linked.

	X-linked transcripts	Autosomal transcripts	Y-linked transcripts
Transcripts carrying fixed differences	434	8	0
Transcripts without fixed differences	1163	4554	23
Proportion of transcripts carrying fixed differences	0.2712	0.0018	0
Total fixed differences	955	11	0

### Drive-Associated Proteins Are Evolving Rapidly

**Figure 3:**
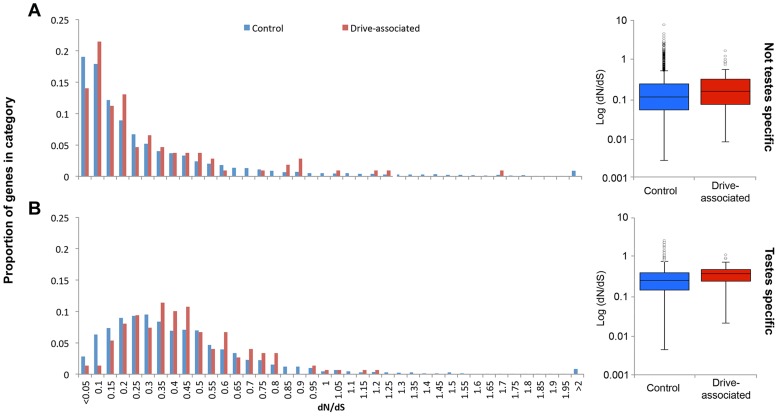
Drive-associated transcripts show elevated rates of protein evolution. (A) Drive-associated transcripts (red) show higher dN/dS, calculated for all transcripts with orthologs in the *T. whitei* testes transcriptome, than controls (blue) and this holds true when (B) only testes-specific genes are compared. Across all genes, testes-specific genes have elevated median dN/dS compared to genes with expression in other tissues (compare A to B).

Because only a small proportion of drive-associated transcripts had *Drosophila* orthologs, and because the taxa diverged ∼70 MYA [51], to assess protein sequence evolution we used *T. whitei*, a closely related species of stalk-eyed fly (∼1.8–3.5 MY since most recent common ancestor [52]) for comparison. RNA was extracted from testes collected from a *T. whitei* lab population derived from flies collected in Chiang Mai, Thailand, sequenced using Illumina Hi-Seq paired-end reads and assembled *de novo* using Trinity. Proteins were predicted from the *T. whitei* transcripts and aligned to *T. dalmanni* predicted proteins. Because a larger proportion of drive-associated than control transcripts are expressed in testes and have moved between the X and autosomes (see above), we used a generalized linear model with an exponential distribution and reciprocal link function to determine if drive association, expression (testis-specific or not), transcript movement, or chromosome location influence protein evolution (Table S3). In the best fitting model, significant factors positively affecting dN/dS included expression (P<0.0001), transcript movement (P = 0.0042), and drive-association (P = 0.0434). Testis-specific genes have elevated dN/dS compared to genes with expression in other tissues (median dN/dS = 0.338 vs 0.155, P<2.2e-16 Mann-Whitney U test). Genes inferred to have moved have lower dN/dS than genes that have not moved (median dN/dS = 0.125 vs 0.136, P = 0.004, Mann-Whitney U test). Drive-associated transcripts show higher dN/dS than controls (Figure 3A, median dN/dS = 0.307 vs 0.199, P = 3.8e-7, Mann-Whitney U test) and this holds true when only testis-specific genes are compared (Figure 3B, dN/dS = 0.379 vs 0.336 P = 0.0284 Mann-Whitney U test).

We conclude that (as expected [53]) testis-specificity influences much of the variation in dN/dS, but in addition, drive-associated transcripts are more likely to be evolving rapidly even after accounting for testis specificity. It is possible that a lack of constraint rather than positive selection is causing the increase in dN/dS among drive-associated transcripts, i.e. weakly deleterious alleles are expected to fix more rapidly when recombination is suppressed, as appears to be the case for large portions of X^SR^ in *T. dalmanni* [37]. If the accumulation of deleterious alleles among X-linked genes due to a lack of recombination, i.e. Muller's ratchet [54], was the main cause of elevation in dN/dS, we would expect X-linked genes to have higher dN/dS than autosomal genes. However, X-linkage did not affect dN/dS in any model (Table S3). The most likely explanation is, therefore, that recent expression divergence in drive-associated transcripts coincides with divergence at the sequence level.

### Fixed Differences Accumulate on X^SR^

**Figure 4:**
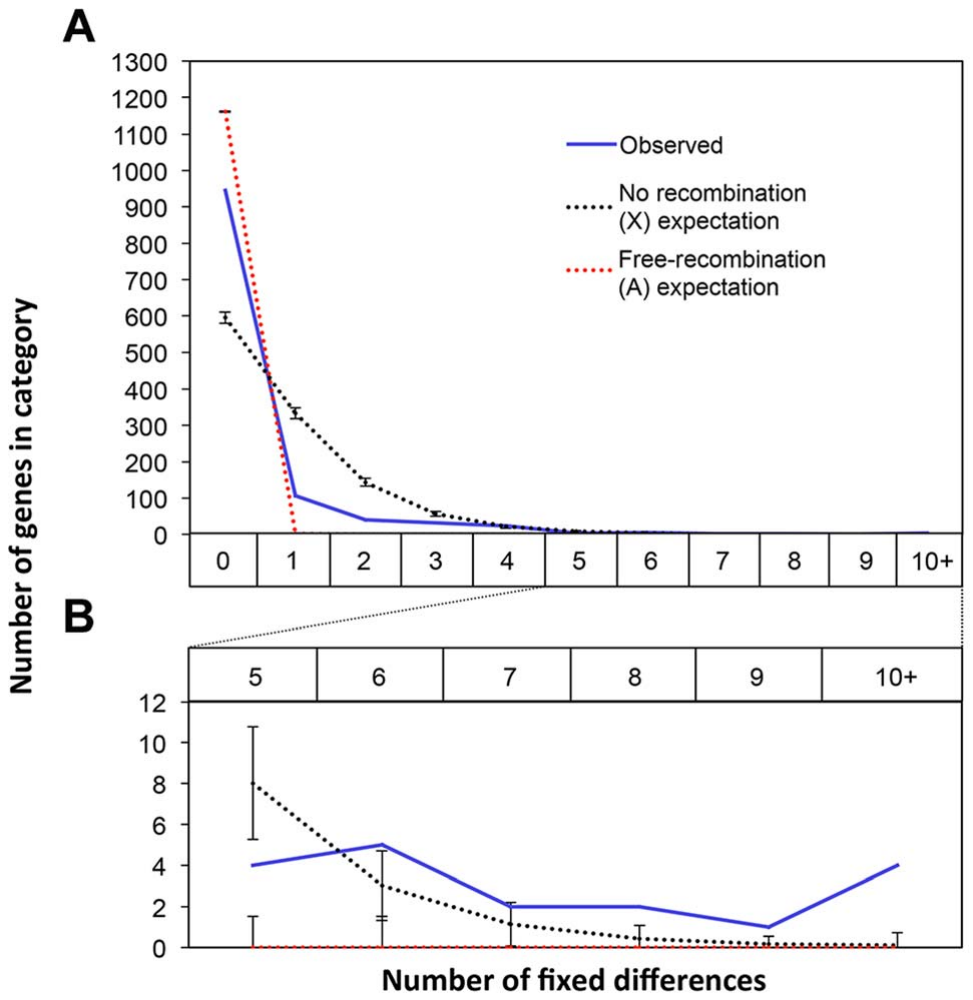
Fixed differences in X-linked genes are not uniformly distributed across the X. There are 955 fixed differences on the X chromosome between the X^SR^ and X^ST^ transcriptomes across 27% of the X-linked transcripts. We compared the observed counts of transcripts with various numbers of fixed differences (blue line) to the expected distribution of fixed differences across genes if fixed differences were distributed randomly across these genes using draws from the binomial distribution with fixed differences appearing at a rate proportional to the observed per-basepair rate of fixed differences on the X (black) or autosomes (red). Compared to the X expectation there was an excess of X-linked transcripts with zero fixed differences (A). There was also an excess of transcripts with six or more fixed differences (B). Together these data suggest that fixed differences on the X are clustered non-uniformly with some transcripts having more fixed differences than expected.

Recombination is suppressed between X^SR^ and X^ST^ in *T. dalmanni* [26]. This is a common feature in several extant drive systems (see [25]) and may have evolved as a way to prevent recombination breaking up suites of genes that are beneficial to individuals carrying drive loci [14]. Recombination suppression leads to accumulation of genetic differences between the suppressed regions and is thought to be the primary mechanism leading to the degeneration of the Y chromosome [54], [55]. We hypothesized that it should therefore be possible to identify fixed genetic differences between the suppressed regions on X^SR^ and X^ST^ using RNAseq data. Conversely, in a freely breeding population there should be very few fixed differences between autosomal genes in X^SR^ and X^ST^ males. Indeed, we found 955 fixed differences in X-linked transcripts but only 11 fixed differences between X^SR^ and X^ST^ males in autosomal transcripts (Table 2). Even more remarkably, roughly one-fourth of X-linked transcripts contain at least one fixed difference. Given the large number of individuals sampled (∼60 for each drive and standard individuals, see methods), this excess of X-linked fixed differences cannot be explained by the fact that the X chromosome was sampled at half the depth of the autosomes (see Figure S2). If the entire X chromosome were nonrecombining, a simple null expectation would be that fixed differences should be randomly distributed across the transcripts based only on their length. We performed a simulation to test this hypothesis. Based on the observed per-basepair frequency of fixed differences in X-linked (6.35 e-04) and autosomal transcripts (2.06 e-06) and the known lengths of all transcripts used in this study, we performed 10,000 draws from the binomial distribution to determine the expected number of genes carrying one or more fixed differences on each type of chromosome. We found more genes with no fixed differences (Figure 4), and more genes with six or more fixed differences (Figure 4, inset) than the X expectation. These data could be interpreted in one of two ways. First, this increased “clustering” of X-linked fixed differences could be due to repeated selection on multiple sites in certain transcripts. Indeed, theory predicts that genes modifying drive would be under positive selection after drive arose [56]. Alternatively, the excess of genes with no fixed differences could be due to free recombination on a relatively large portion of the X chromosome, either currently or historically. For example, if the drive X chromosome slowly accumulated multiple inversions in order to become fully non-recombining, then that could explain the presence of fewer fixed differences if some of the inversions are more recent than others.

### Potential Functions of Drive-Associated Transcripts

Observed differences in transcription may be the direct result of genetic changes responsible for meiotic drive, or may impact other functions through linkage to the drive locus. While many drive-associated transcripts are expressed in the testis and hence may be directly involved in drive, others have higher expression in other tissues. To further understand what functions drive-associated transcripts might have, we first used the DAVID functional analysis tool [57] to determine whether drive-associated transcripts with Drosophila orthologs were enriched for any gene ontology (GO) terms. We found that at a 5% FDR cutoff, four ion binding GO terms (GO:0008270, GO:0043169, GO:0043167, and GO:0046872) were enriched among drive-associated transcripts (Table S4). None of these terms were enriched in the control gene set, despite the fact that the control genes are a much larger sample giving increased power. The genes in these GO categories were functionally diverse, and included a cytochrome P450, *calmodulin*, *chiffon* (an eggshell protein), and many others. In total, 37 drive-associated transcripts had at least one significant GO term (Table S4).

In *T. dalmanni* sperm bundles from drive males contain approximately 50% arrested sperm [33]. The molecular mechanism leading to arrest is not known, but inspection of spermatid bundles indicates that Y-bearing sperm fail to complete elongation in drive males. In one example of sex chromosome meiotic drive in *Drosophila melanogaster* the Y-sister chromatids fail to segregate during meiosis II, ultimately leading to arrest of Y sperm development prior to elongation [58]. It may also be the case that Y-bearing sperm undergo apoptosis or another form of regulated cell death. Among drive-associated transcripts, we found several with Drosophila orthologs involved in centrosome function, meiosis, mitosis, fertility, and apoptosis (Table S5). These genes may be causal to drive, or they may be misregulated due to the action of upstream drive genes. We also found several genes that are important to male and female fertility in Drosophila. *Fs(1)N* causes sterility in females when lost [59], *Tom7* [60] and *Hexo1* [61] are involved in sperm transfer and spermatogenesis respectively, and the loss of *tj* (traffic jam) causes sterility in both sexes [62]. Interestingly, one group of drive-associated transcripts are known to affect brain and eye development in *D. melanogaster*. Misregulation of these genes – if extended to development - could underlie some of the traits associated with drive [28], such as changes in behavior and eye span (Table S5). Previously, we identified a group of genes differentially expressed in *T. dalmanni* males selected for longer and shorter eyespan [63]. Two of these genes, *chiffon*, and *CG4598* were also drive-associated and may be involved in the genetic link between shorter eyespan and meiotic drive [64]. *Chiffon* has a variety of functions, one of which is exon guidance in photoreceptors [65], *CG4598* is a member of the Crotonase subfamily and is of unknown function.

Finally, genes that have differences in expression may be good candidates for the proximal causes of meiotic drive and associated phenotypes, but a heritable difference in sequence is required to trigger drive. As a first attempt to identify possible candidate genes, we identified a subset of X-linked, drive-associated transcripts that contained fixed differences between X^SR^ and X^ST^. We determined whether these fixed differences fell into the protein-coding regions or the UTRs of genes, and whether they were synonymous or nonsynonymous if protein-coding. We identified 24 drive-associated transcripts (of 46 drive-associated transcripts carrying fixed differences) that carried at least one nonsynonymous fixed difference between X^ST^ and X^SR^ (Table 3). Many of these genes are also evolving fairly rapidly between *T. dalmanni* and *T. whitei*, with dN/dS values well above the average for all genes, though not necessarily due to positive selection (i.e. dN/dS is not >1). While most of these genes are testes specific (Tau is >0.95), six of 24 fall into the ovary and testis expression category, implying they could function in both male and female reproduction. A gene called *klarsicht* also contains two nonsynonymous fixed differences and reduced expression in X^SR^ testes. This gene – a transport regulator - has been linked to a variety of functions including eye development [66]. Interestingly, it was recently discovered that *klar* mutants affect nonrandom segregation of sister chromatids in germline stem cells of the testis [67]. While *klar* mutants did not affect segregation of chromosome pairs, the association with nonrandom chromosome segregation is intriguing and worthy of future investigation.

**Table 3:** Drive-associated transcripts carrying nonsynonymous fixed differences.

	log2 (X^ST^/X^SR^)	dN/dS vs. *T. whitei*	*D. melanogaster* gene name	Drive/standard fixed differences				Tau	Expression pattern
				Total	S	NS	UTR		
comp147722	−1.96	0.0675	*santa-maria*	3	1	2	0	0.8513	Larva
comp158645	−1.54	0.0295	*osm-6*	1	0	1	0	0.9617	Testis
comp162098	−0.8	0.3222	*CG10431*	10	4	4	2	0.7904	Ovary & testis
comp162893	−0.78	0.4022	*Kif3C*	2	1	1	0	0.9230	Ovary & testis
comp159374	−0.73	0.3437	*Fen1*	1	0	1	0	0.7962	Ovary & testis
comp152671	−0.61	0.2556	*CG16890*	3	1	1	1	0.3936	Ovary
comp156565	0.5	0.0693	*Dap160*	4	3	1	0	0.8510	Ovary & testis
comp162851	0.54	0.5034	*klar*	2	0	2	0	0.9990	Testis
comp151771	0.68	0.1727	*capu*	2	1	1	0	0.9236	Testis
comp153672	0.71			5	2	1	2	0.9978	Testis
comp151770	0.71	0.2461	*CG7810*	1	0	1	0	0.6293	Ovary & testis
comp135354	0.76	0.1107	*CG9004*	5	2	3	0	0.7418	Ovary & testis
comp157383	0.8	0.1611	*CG13855*	2	1	1	0	0.9867	Testis
comp156142	0.8			1	0	1	0	0.9937	Testis
comp152380	0.87	0.5950	*Rack1*	4	1	1	2	0.9910	Testis
comp158877	0.88	0.6065		1	0	1	0	0.9966	Testis
comp160201	0.93	0.3506	*ppk11*	2	1	1	0	0.9963	Testis
comp157815	0.94	0.4940		1	0	1	0	0.9959	Testis
comp149172	0.95			3	1	2	0	0.9957	Testis
comp161993	1.1	0.5234		4	1	3	0	0.9946	Testis
comp130127	1.26	0.1705		4	0	1	3	0.9922	Testis
comp151215	1.59	0.3789		1	0	1	0	0.9853	Testis
comp152096	1.73	0.0586	*CG3645*	3	2	1	0	0.9045	Testis
comp156183	1.94	0.5882		2	1	1	0	0.9895	Testis

## Discussion

Although distortion of sex ratio due to meiotic drive has been observed in a variety of species for over 50 years [46], [68]–[73], the genetic causes of sex chromosome drive remain obscure in the vast majority of cases. Sex chromosome meiotic drive is notoriously recalcitrant to traditional genetic dissection due to its tendency to associate with chromosomal inversions, presumably as a result of meiotic drive involving the combined action of multiple loci [4], [69], [74]. In addition, X-chromosome drive is predicted to have consequences for processes ranging from sexual selection to the evolution of the genome. As populations become biased towards one sex or the other, inter- and intra- sexual selective pressures diverge. As females become increasingly common and if male reproduction is at all costly, males may become choosy [75]. Meanwhile, females employing strategies that increase their chances of mating with a standard male would benefit, as more of their offspring would be the rare (male) sex [2]. This might occur through female preference for a linked trait [34] or multiple mating [76]. Meanwhile, sex-ratio meiotic drive is expected to favor Y-linked and autosomal alleles that suppress drive, subjecting the genome to strong local selection pressures. Fixation of alleles causing or modifying drive may be nonadaptive or even maladaptive.

To gain insight into the genetic differences between nonrecombining drive and standard X chromosomes, we used RNAseq to measure differences in expression between drive and standard testes from a species, *T. dalmanni*, with high frequencies of unsuppressed X chromosome meiotic drive and a wealth of biological data associated with the drive system. We sequenced testes from males carrying X^SR^ and standard X chromosomes and identified a group of genes that are significantly differentially expressed, including a number of candidate genes whose *D. melanogaster* orthologs have been associated with male sterility and chromosomal nondisjunction during mitotic and meiotic divisions, and some of which carried fixed differences. While some of these genes may have diverged in expression due to neutral processes associated with sequence divergence of X^SR^ from standard X chromosomes, others may either impact, or be impacted by meiotic drive directly. Interestingly, some of the genes whose expression changed in X^SR^ males are also strongly expressed in other tissues and may be involved in other observed phenotypic differences between drive and non-drive males, including genes that may be involved in differences between drive and standard males in the sexually selected exaggerated eye stalk phenotype [64]. X^SR^ males are generally at a reproductive disadvantage as they are less able to directly compete with other males for matings due to reduced ornament size [64] and for fertilizations after copulation due to weaker sperm competitive ability [32]. Conversely, heterozygous female carriers of X^SR^ have higher fecundity than their X^ST^ sisters [28]. It has been suggested that the overdominant effect of X^SR^ on female fecundity may be one reason why drive is still expressed in the population, rather than being suppressed as in many *Drosophila spp*. We found that drive-associated transcripts were enriched for genes showing elevated expression in both the testis and ovary. If loci impact both drive in males and fecundity in females, natural selection may select against suppression of the activity of these genes. In fact, models of drive demonstrate that in the absence of frequency dependent selection, a stable drive polymorphism may still be maintained when female fecundity and drive are impacted by the same locus, or tightly linked loci [6], [46]. Due to the relative scarcity of genes that impact both male and female reproduction [77], it has been thought unlikely that the same locus would impact both traits [14]. The excess of drive-associated genes expressed in both tissues provides a counter example that warrants further investigation.

In addition to identifying specific candidate genes that may be involved in meiotic drive in *T. dalmanni*, we identified a number of patterns associated with genes that are differentially expressed between X^SR^ and X^ST^ testes. First, we found that the X chromosome carried a majority (∼80%) of the genes whose expression differed between X^SR^ and X^ST^ testes. In addition, we found that there was a large excess of gene movement from the autosomes to the X chromosome relative to Drosophila, especially among testes-specific genes, though this type of movement was enriched in control genes as well as drive-associated transcripts (Figure 2). Inheritance of X^SR^ is generally sufficient to induce drive regardless of the genetic background, implying that segregating suppressors of X^SR^ are absent or rare in nature. However, although the X^SR^ chromosome has a strong genetic effect to induce meiotic drive, it is not necessarily obvious that changes in expression should be limited to the X chromosome. Meiotic drive genes on X^SR^ could in principle act as “triggers” that alter expression of genes in *trans* across the genome. Alternatively, *cis* regulatory mutations, copy number changes, and the accumulation of null alleles [54] could affect the expression of genes on X^SR^ directly. Our finding of a large X effect on drive-associated expression, along with the accumulation of many fixed genetic differences between X^SR^ and X^ST^ genes, suggests that *cis* effects dominate *trans* effects in the case of sex chromosome meiotic drive. This is consistent with the hypothesis that stable persistence of a sex chromosome drive polymorphism requires that a suite of co-adapted genes be inherited together, often in the form of a large inversion or series of inversions [14]. Another possibility is that a meiotic drive trigger gene could impact expression preferentially on the X chromosome (chromosome-specific gene regulation). This is seen in the *Slx/Sly* system in mice, although in that case sex-linked genes are either up- or down-regulated by SLX or SLY respectively rather than causing a variety of expression changes (Coquet et al 2012).

We also found that these genes are evolving more rapidly at the protein level (dN/dS), and this increased evolutionary rate could not be entirely explained by a tendency of these genes to be testes-specific or linked to the X chromosome. By virtue of violating Mendelian inheritance, drive alleles produce a strong local fitness advantage, and if not suppressed, are expected to increase in frequency in the population, both removing polymorphism and bringing hitchhiking variants with them [1]. It is possible that much of the acceleration in the rate of protein evolution we observe is due to relaxed purifying selection during such a sweep (see [78], [79]). Alternatively, as X^SR^ reaches higher frequency in the population, other genes in the genome may begin to evolve to adapt to the new genetic context. Theory predicts, for example, rapid evolution of modifier and suppressor loci should occur [56], [80]. Although we have not previously identified these loci, it is plausible that some differentially expressed loci may be modifiers of drive.

Because the testes we collected were from an outbred population, we were able to use natural variants in the X^SR^ and X^ST^ individuals to confirm that X^SR^ almost certainly contains at least one inversion that prevents genetic exchange between the X^SR^ and X^ST^ chromosomes. Nearly 1,000 variants have become fixed between X^SR^ and X^ST^, whereas only 11 such differences exist between autosomes carried by X^SR^ and X^ST^ males. It would be difficult to explain this discrepancy in any way other than a lack of genetic exchange between X^SR^ and X^ST^ – it is highly unlikely that freely recombining chromosomes would pick up any fixed differences, whether X-linked or autosomal (Figure S2). A simple simulation (Figure 4) demonstrates that there are more genes carrying zero fixed differences than expected if recombination was suppressed uniformly across the X chromosome and affected all genes equally. The apparent clustering of fixed differences could be due to some proportion of the drive X chromosome continuing to recombine normally with standard X chromosomes. Alternatively, the fixed differences may cluster due to selection acting on certain genes differently between drive and standard individuals, even when the entire drive X is failing to recombine with standard X chromosomes. Further genetic analysis will be needed to discover which regions of the X^SR^ chromosome recombine and which do not. A number of these fixed differences caused nonsynonymous changes in proteins, some of which were drive-associated (Table 3). These genes may be good initial targets for future analysis.

Finally, a large number of genetically isolated populations of *T. dalmanni* – as well as the closely related species *T. whitei* - can be found in southeast Asia and the valleys neighboring the Gombak valley from which the flies used for this study were collected. Many of these populations express sex chromosome drive ([81] and unpublished data). Although reverse genetic dissection is difficult in this species, these flies represent a potential natural laboratory for the study of gene expression and meiotic drive. Sex chromosome drive has persisted as a stable polymorphism in *T. dalmanni* for many generations – possibly for millions of years, given that it exists in the sexually dimorphic sister species in the same genus. Within such a long timescale, drive X chromosomes may have arisen once, or they might be evolving constantly through arms races between suppressors and drivers. In either case, further study of teleopsid populations and species will advance our understanding of how meiotic drive can impact gene structure and function when it is a constant evolutionary companion.

## Methods

### Sample Collection and Determination of X^SR^ and X^ST^ Genotype

Testes for RNAseq were dissected from mature *T. dalmanni* males derived from an outbred lab population established in 1999 (cf. [28]). This population was founded from ∼100 flies caught in the Gombak valley in Malaysia and was maintained for approximately 30–40 overlapping generations at that size. After dissection, testes were transferred to RNAlater and stored at −20°C, and remaining tissue was used to extract DNA using Chelex [82]. We determined X^SR^/X^ST^ status using three X-linked microsatellite markers previously associated with meiotic drive [26], [28]. These three markers span the X, and previously a “drive” haplotype including these three loci was diagnostic for drive [37]. The frequency of drive in the 1999 population was estimated to be 24% (15/62 phenotypically screened flies) in 2003 [28] and 18% (22/122 genotypically screened flies) in 2010. Multiplexed PCR was performed using three fluorescently labeled primers and PCR products were genotyped on an ABI 3730×l DNA analyzer (Applied Biosystems). Products were sized using Rox500 and scored with GeneMapper 4.0 software (Applied Biosystems). Two replicate samples each were pooled for individuals carrying X^SR^ or X^ST^ as follows: sample X^SR-^1, 35 testes pairs; X^SR-^2, 30 testes pairs; sample X^ST-^1, 38 testes pairs; and sample X^ST-^2, 30 testes pairs. RNA was extracted using the mirVana RNA Isolation Kit (Invitrogen) according to manufacturer's protocols for extracting mRNA. Samples were sent to Cofactor Genomics (St. Louis, MO) for bar-coding and library preparation and 51.5 million 60 bp paired-end reads were obtained by sequencing all four libraries across two lanes in an Illumina Genome Analyzer run.

### Tissue Transcriptome Assembly

A *T. dalmanni* draft transcriptome assembly was generated using 100 bp paired end Illumina HiSeq reads from five *T. dalmanni* tissues (ovaries, testes, gonadectomized females, gonadectomized males, and third instar larvae), 84 bp paired end Illumina GA reads from female and male heads, and the 60 bp X^ST^ and X^SR^ testes paired reads described above. Together, these samples produced ∼308.5 million reads and ∼55.5 Gbp of sequence. All reads were assembled into a single transcriptome using Trinity (paired end mode, –CPU 24, –kmer_method inchworm –max_memory 190G). The resulting transcriptome assembly and associated raw read data can be obtained from NCBI as BioProject accession PRJNA240197. In order to be compliant with NCBI's TSA (transcriptome sequence assembly) database, a small number of the contigs in the original assembly were trimmed to remove potential vector contaminants, and a handful of contigs were shorter than the minimum 200 bp required for TSA and could not be uploaded. These sequences are available from the authors by request. Details of sequencing and assembly can be found in Table S6.

### Identifying Genes Differentially Expressed between X^SR^ and X^ST^ Samples

We used a modified version of bwa [38] that allows multiple mapping (available as part of the Trinity RNAseq software bundle, [83] trinityrnaseq.sourceforge.net) to align the left end reads back to the transcriptome. We chose to align the left end only because 1) the right end is not independent from the left and therefore adds no additional power to the analysis and 2) the first read is typically higher quality than the second [84]. The *T. dalmanni* transcriptome assembly contains many genes that are represented by multiple transcripts - often, these are multiple isoforms of the same gene. After alignment with bwa, expression was quantified using RSEM to correct for hits to multiple isoforms of the same gene. Genes were defined as those transcripts derived from the same Trinity component (see trinityrnaseq.sourceforge.net), and read counts were corrected at the gene/component and isoform/seq level based on the share of reads derived from each isoform. Corrected gene-level read counts were used with DESeq [40] to identify significantly differentially expressed genes between the two X^SR^ and two X^ST^ samples using a 0.001 FDR cutoff and using DESeq's independent filtering option to improve power. The highest expressed isoform for each gene/component was identified and used for subsequent analyses. To ensure our results were independent of the statistical method, we also used edgeR [85] to identify significantly differentially expressed genes and obtained qualitatively similar results (Table S7). Only DESeq results are presented henceforth.

### Expression Analysis

The expression patterns of *T. dalmanni* genes were assessed using transcriptome sequencing from six *T. dalmanni* tissues (ovaries, testes, gonadectomized females, gonadectomized males, adult heads, and third instar larvae). With the exception of the heads, each of these tissues included two biological replicates. For the heads, one sample was from females and the other was from males. These were treated as biological replicates for the analysis of expression across tissues as we were more interested in differences between tissues than between the sexes *per se*. Corrected read counts for each sample were obtained as described for the X^SR^ *versus* X^ST^ comparison above. Normalized gene-level expression values (FPKM) were determined and expression profiles were assessed using tools provided with the trinity RNAseq package [83] as described on trinityrnaseq.sourceforge.net, “Identifying Differentially Expressed Transcripts” (see also, [43]). A 0.001 FDR cutoff was used to identify genes that were significantly differentially expressed between samples. The significantly differentially expressed genes were then grouped by similarity of their expression patterns using Euclidean complete clustering. Next, we used k-means clustering to define distinct expression pattern groupings from among the differentially expressed genes (see Figure S1). We tried a range of K values (6 to 12) and assessed the number of genes and the expression profile for each cluster. We chose K = 8 for further analysis, as this number of clusters provided the maximal number of qualitatively different expression patterns. Increasing the cluster number to 9 added a cluster with different expression levels but the same expression pattern as already represented by previous clusters. In addition to the gene expression pattern analysis presented above, we also calculated a measure of tissue specificity – Tau [44] – for each gene using the average of the two FPKM values for each tissue. Genes with Tau >0.95 were considered to be expressed specifically in the highest expressed tissue. For all subsequent analyses using the sequence of a gene (gene prediction, orthology prediction, linkage, etc.), the highest expressed isoform (Trinity variant) of each gene was used as the representative sequence.

### RT-PCR Confirmation of Gene Expression Variation

Testes were dissected from a newly collected (August, 2012) population of *T. dalmanni* Gombak. This population was used because genotyping of ∼150 flies in the 1999 population failed to identify any males carrying the previously defined X^SR^ haplotype [28]. To obtain testes from drive males, we genotyped second-generation *T. dalmanni* males from the 2012 Gombak population using the three markers described above. A male was defined as carrying a drive haplotype if he carried an ms125 allele <152, an ms244 allele >238, and an ms395 allele >230. Breeding studies using the 2012 population confirm that males with these haplotypes produce drive and reveal that other drive haplotypes exist (unpublished data). To confirm standard status we phenotyped individuals for unbiased progeny sex ratios (between 0.4 and 0.6 proportion sons, 50 + progeny). RNA was extracted from pools of 3 testes pairs using the mirVana kit (Invitrogen AM1560) and first strand cDNA was synthesized using M-MLV reverse transcriptase (Promega M1705). From the list of candidate genes, 18 (11 that were up in X^ST^ testes and 7 that were up in X^SR^ testes) were selected for confirmation by quantitative reverse transcription polymerase chain reaction (qRT-PCR). qRT-PCR was conducted on a Bio-RAD CFX real-time PCR machine using SYBR 2× RT-PCR mix (Invitrogen 4472942), 1 uL of cDNA template and gene specific primers. In order for a primer pair to be used, it had to have a Ct value below 32 in both replicates in at least of one of the two conditions, otherwise we discarded it from the analysis. Six genes were excluded using this criterion. A failure to detect expression in RT-PCR could be for a variety of reasons: 1) the number of testes in the pool was much smaller so if there was an expression polymorphism in the original pools it might have been missed, 2) the population sampled for qRT-PCR was 12 years separated from the lab population so differences in expression may be present, and 3) the total amount of RNA was much less. Primers were also tested on genomic DNA to ensure that failure to amplify was not due to primer failure. Expression was quantified relative to a control gene (GAPDH-2), and when all four samples showed robust expression, a t-test was performed on resulting delta Ct values between the two conditions. When data was available from all samples, the log_2_ expression differential was calculated using the delta-delta Ct method [86] between X^SR^ and X^ST^ samples relative to GAPDH-2 (Table S1).

### Control Genes

After determination of expression values for all genes (above), we created a control gene list by removing the most weakly expressed genes in the Trinity assembly. This step prevents misinterpretation of results that could arise from inclusion of very weakly expressed transcripts in the control dataset (such weakly expressed transcripts could never be detected as drive-associated due to a lack of statistical power). Therefore, we defined an expression floor using the drive-associated genes. We identified the tissue for each drive-associated gene that had the highest expression level (the maximum expression level for that gene) and ranked genes by this value from lowest to highest. We used the lowest maximum expression level among the drive-associated transcripts (FPKM = 0.86) as the expression cutoff for the control gene set. If the highest expressed sample for a transcript had an FPKM>0.86, it was included in the control gene set. Otherwise, it was removed from further analysis.

### Annotation of *D. melanogaster* Orthologs

Transcripts were annotated as having Drosophila orthologs using blastp. First, proteins were predicted from *T. dalmanni* transcripts using two methods – the longest start to stop ORF and FrameFinder [87], which can find longer ORFs if a transcript is truncated due to poor assembly. FrameFinder was run with the local (not strict) model using a word probability set generated from the entire *T. dalmanni* transcriptome using the Fasta2count and wordprob tools included with FrameFinder, and options were set to disallow frameshifts and indels (options: -I −500 –D −500 –F −500 –s False). The proteins generated by these two methods for each transcript were aligned by blastp to the *D. melanogaster* proteome (Flybase v. 5.50) and the best hit in *T. dalmanni* was kept for each gene. An e-value cutoff of 0.1 was used, and only hits covering 50% or more of the *D. melanogaster* protein were kept. The coverage cutoff prevented keeping partial hits due to the assembly incorrectly splitting a gene into two contigs, both contigs hitting different parts of the same ortholog, and being seen, incorrectly, as paralogs. If both the framefinder and the longest orf predictions had qualifying hits, the best hit (by e-value, then by %ID) was kept. In the case of a tie, the FrameFinder hit was kept. These protein hits were annotated using the Flybase batch download tool. The gene family size in *T. dalmanni* was estimated for genes with *D. melanogaster* orthologs. The number of occurrences of each Flybase gene ID (Fbgn) among the putative orthologs of the control gene set was used to estimate the *T. dalmanni* gene family size for each gene.

### Annotation of *T. whitei* Orthologs and Estimation of dN/dS

A *T. whitei* transcriptome was assembled using Trinity [83] (–max_memory 190G –CPU 24 –kmer_method inchworm, paired-read mode) on RNAseq from a pool of approximately 30 pairs of *T. whitei* testes (33,753,826 100 bp paired end reads were generated, and 60,650 contigs were assembled). The *T. whitei* assembly and raw data can be obtained from the NCBI website under the BioProject accession PRJNA241109. As with the *T. dalmanni* assembly, to be compliant with NCBI's TSA database, a small number of the contigs in the assembly were trimmed to remove potential vector contaminants and sequences shorter than 200 bp were removed. These sequences are available from the authors by request. Proteins were predicted from the resulting transcriptome assembly as with *T. dalmanni* using both FrameFinder and the longest ORF as described above (the *T. whitei* transcriptome was used to create a word probability set for FrameFinder prediction). The *T. whitei* proteins were aligned by blastp to the predicted *T. dalmanni* proteins and hits with e values <0.1 were kept. For each gene, whichever predicted protein had the best hit to a predicated *T. whitei* protein was kept. The resulting *T. whitei* and *T. dalmanni* protein pairs were aligned using Clustal omega [88], and the consensus transcriptome sequences were mapped onto the protein alignments (after trimming excess sequence). dN/dS was predicted from each alignment greater than 50 amino acids in length using SNAP [89]. Only consensus sequences were used in calculating dN/dS - polymorphism in the RNAseq data was not considered in this analysis, as such data from RNAseq data can be unreliable (high or low levels of coverage due to differences in gene expression may cause over or underestimation of the number of polymorphic sites, respectively).

### GO Analysis

Gene ontology analysis was performed for Drosophila orthologs using DAVID functional annotation tools (http://david.abcc.ncifcrf.gov, [57], [90]). The list of *D. melanogaster* orthologs to drive-associated transcripts was compared to the orthologs in the control gene list and to the entire *D. melanogaster* proteome using DAVID's functional annotation tables tool. Annotations with an FDR<0.05 were considered significant when interpreting the output.

### Analysis of Gene Linkage Relative to *Drosophila melanogaster*

We used data from comparative genomic hybridization (CGH) to determine linkage of genes differentially expressed between drive and standard samples as well as the rest of the Trinity assembly. The CGH data are accessible using accession number GSE55601 from the NCBI Gene Expression Omnibus (GEO). First, the log_2_ ratio of female to male expression was calculated for each probe on each of four duplicate oligonucleotide Agilent arrays containing 180K probes representing 12,000 unique genes. These values were normalized so that the maximum number of probes had a log_2_(f/m) ratio of 0, the expected value given the nature of the array (divergence from 0 is caused by small differences in the quality or quantity of genomic DNA from the two sexes applied to the array). The sequences for the probes (see GSE55601) were then aligned with BLAT (multiple matching allowed, perfect hit required) to the Trinity assembly, giving a set of probes matching each transcript. The median of the probe values for a given transcript was calculated for each array, and then a median and a 95% confidence interval (CI) was calculated across the four arrays. Calls for the linkage of each contig were as follows: 1) if the upper bound of the CI was less than −2, the transcript was called Y-linked; 2) if the lower bound of the CI was greater than 0.5, the transcript was called X-linked; 3) if the CI was entirely between −2 and 0.5, the transcript was called autosomal; 4) if the CI overlapped any of these bounds, or if a transcript had only a single probe or a single array informing on it, it was called U. For genes with putative *D. melanogaster* orthologs, the linkage of each gene was compared relative to *D. melanogaster*. The X chromosome in *T. dalmanni* is mostly orthologous to chromosome 2L in *D. melanogaster* [30]. Therefore, genes that are X-linked in *T. dalmanni* and on non-2L chromosomes in *D. melanogaster* have most likely moved relative to one another in one of the two lineages. Likewise, autosomal genes in *T. dalmanni* that are on 2L in *D. melanogaster* have most likely moved at some point since they last shared a common ancestor.

### Estimates of Genetic Differentiation in X^SR^ and X^ST^ RNA Samples

After alignment of the RNAseq data to the transcriptome with bwa, SAMtools [91] was used to create pileup files across the *T. dalmanni* transcriptome. Using the pileup files from X^ST^ and X^SR^ testes, we counted the number of sites on each transcript that were fixed as different alleles between the two samples. We ignored sites that were polymorphic in either the X^ST^, X^SR^, or both samples. In order for a site to be used, it had to have at least 10 reads informing on it in both samples (“10× coverage”), and there had to be at least 100 sites from a given transcript with sufficient read coverage for that transcript to be used. To determine if an excess of fixed differences on the X could be due to the fact that half as many X chromosomes as autosomes are sampled in males, we used fastsimcoal2 [92] to simulate populations of chromosomes with 100,000 SNPs using various values of N_e_, a per SNP recombination rate of 10^−5^ per generation, and a minimum possible derived allele frequency of 10^−6^. We simulated 100 replicate populations with each parameter set. In each simulation we took two samples of equal size from each set of chromosomes, counted the number of fixed differences between the two samples, and then averaged across each set of parameters. Under all parameter sets, once at least 16 chromosomes were sampled, no fixed differences were observed (Figure S2). To determine if the entire X chromosome is nonrecombining, we used the observed probability of a fixed difference per basepair and performed 10,000 simulated draws from the binomial distribution for each of the transcripts that carried a fixed base using the observed distribution of transcript lengths and the per site rates of fixed differences in X-linked and autosomal genes. We compared the resulting distribution of fixed differences per transcript to observed values to determine if the observed distribution was different from that expected if fixed differences are randomly distributed across the X assuming the X chromosome carried by X^SR^ individuals was entirely nonrecombining.

## Supporting Information

Figure S1 Significant patterns of differential expression across tissues. K-means clustering was used to establish qualitatively distinct expression patterns of transcripts across the six sequenced tissues (ovary, testis, larva, heads, male carcass, and female carcass), each with two replicates. Differentially expressed transcripts were clustered into 8 expression pattern clusters as described (methods). The number of genes in each cluster is shown above each plot. Grey lines indicate an individual gene's expression pattern, while the blue marker indicates the mean expression level for a cluster in a given sample.
(TIF)

Figure S2 Zero fixed differences between samples with pool sizes greater than eight. We used fastsimcoal2 to generate samples of 500 chromosomes, each containing 100,000 single nucleotide polymorphisms. These chromosome samples were drawn from simulated populations with various values of Ne, ranging from 100 to 1,000,000 (across top) – 100 independent samples were generated for each value of Ne. The recombination rate between SNPs was constant (10^−5^) and the minimum possible frequency of the derived allele was set to 10^−6^. From the 500 chromosome samples, pairs of smaller samples were drawn randomly to simulate pools drawn from various numbers of individuals (pool sizes 2–16 shown), and the number of fixed differences between the pools was counted. Once at least 16 individuals were sampled from a pool, the likelihood of finding a fixed difference was found to be zero. When Ne was small, there was more variability in the number of fixed differences for the smaller pool sizes.
(TIF)

Table S1 RT-PCR confirmation of drive-associated expression.
(XLSX)

Table S2 Logistic regression models of gene movement.
(XLSX)

Table S3 Generalized linear model of dN/dS.
(XLSX)

Table S4 Results of gene ontology analysis.
(XLSX)

Table S5 Transcripts having Drosophila orthologs with relevant phenotypes.
(XLSX)

Table S6 Transcriptome sequencing and assembly details.
(XLSX)

Table S7 Comparison of EdgeR and DESeq results.
(XLSX)
