# Supplementary figures and images for: Meiotic Drive Impacts Expression and Evolution of X-Linked Genes in Stalk-Eyed Flies
Source: PLoS Genet. 2014 May 15;10(5):e1004362. doi: 10.1371/journal.pgen.1004362 (PMC4022487; doi:10.1371/journal.pgen.1004362)

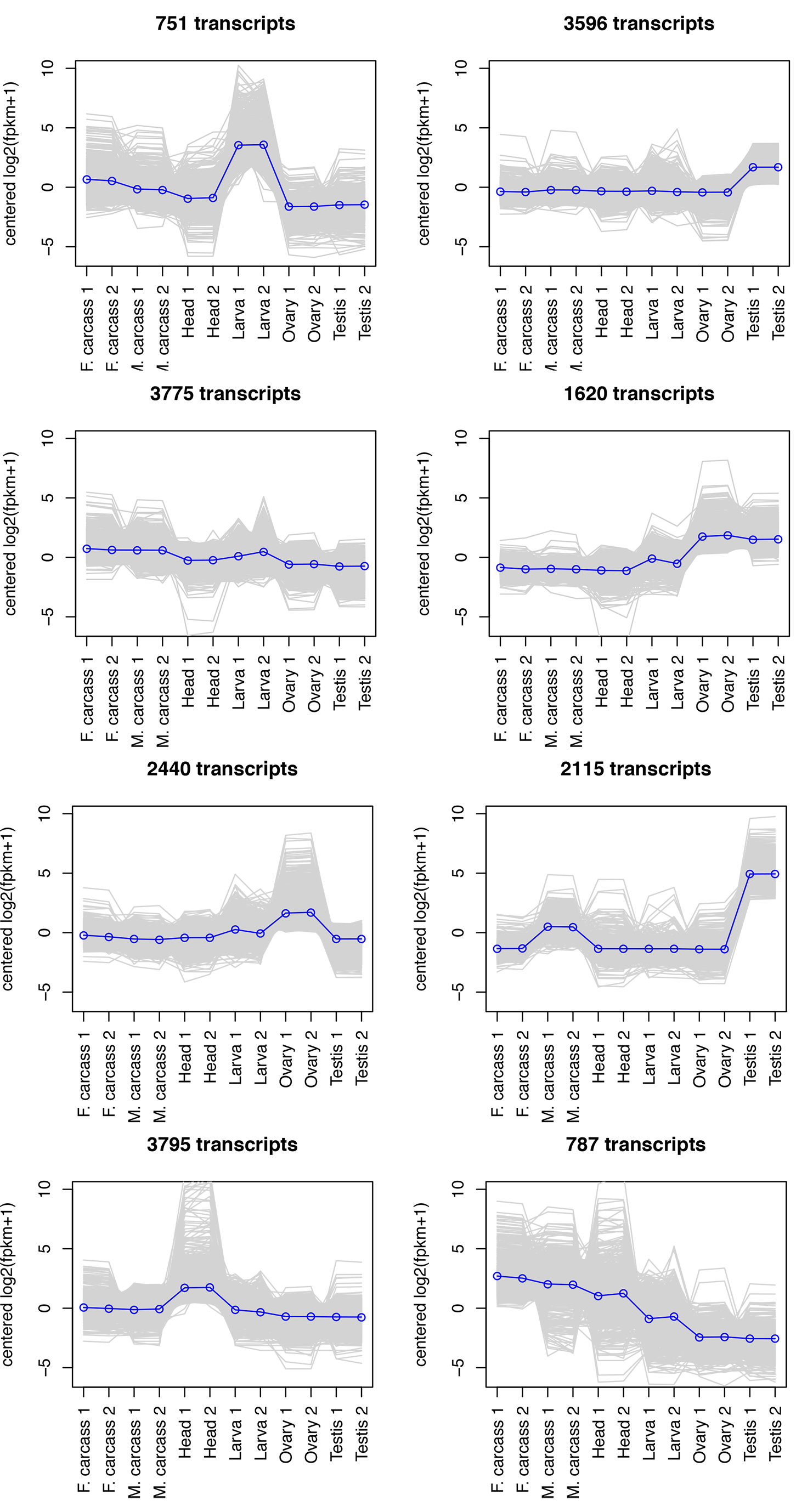

Supplement: Figure S1 — Significant patterns of differential expression across tissues. K-means clustering was used to establish qualitatively distinct expression patterns of transcripts across the six sequenced tissues (ovary, testis, larva, heads, male carcass, and female carcass), each with two replicates. Differentially expressed transcripts were clustered into 8 expression pattern clusters as described (methods). The number of genes in each cluster is shown above each plot. Grey lines indicate an individual gene's expression pattern, while the blue marker indicates the mean expression level for a cluster in a given sample. (TIF) [file pgen.1004362.s001.tif]

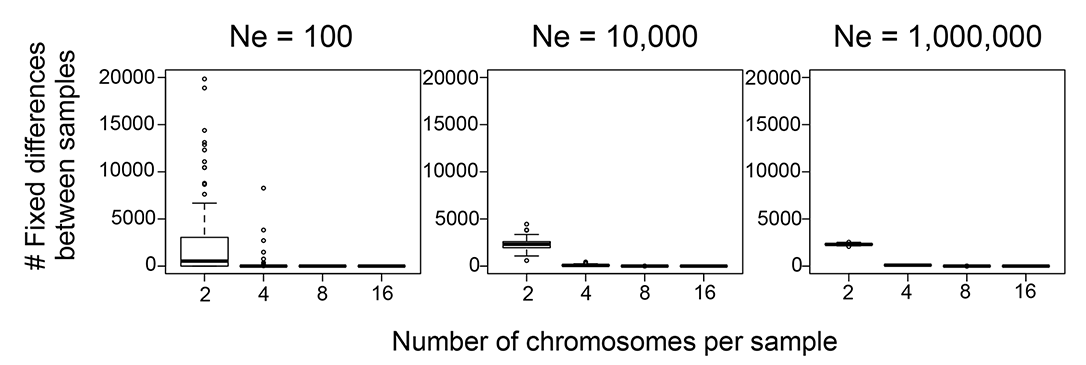

Supplement: Figure S2 — Zero fixed differences between samples with pool sizes greater than eight. We used fastsimcoal2 to generate samples of 500 chromosomes, each containing 100,000 single nucleotide polymorphisms. These chromosome samples were drawn from simulated populations with various values of Ne, ranging from 100 to 1,000,000 (across top) – 100 independent samples were generated for each value of Ne. The recombination rate between SNPs was constant (10−5) and the minimum possible frequency of the derived allele was set to 10−6. From the 500 chromosome samples, pairs of smaller samples were drawn randomly to simulate pools drawn from various numbers of individuals (pool sizes 2–16 shown), and the number of fixed differences between the pools was counted. Once at least 16 individuals were sampled from a pool, the likelihood of finding a fixed difference was found to be zero. When Ne was small, there was more variability in the number of fixed differences for the smaller pool sizes. (TIF) [file pgen.1004362.s002.tif]
